# Reliability, construct validity and measurement potential of the ICF comprehensive core set for osteoarthritis

**DOI:** 10.1186/1471-2474-12-255

**Published:** 2011-11-08

**Authors:** Yeşim Kurtaiş, Derya Őztuna, Ayşe A Küçükdeveci, Şehim Kutlay, Meliha Hafiz, Alan Tennant

**Affiliations:** 1 Ankara University Faculty of Medicine, Department of Physical Medicine & Rehabilitation, Ankara, Turkey; 2 Ankara University Faculty of Medicine, Department of Biostatistics, Ankara, Turkey; 3 The University of Leeds, Faculty of Medicine and Health, The General Infirmary at Leeds, Leeds, UK

## Abstract

**Background:**

This study aimed to investigate the reliability and construct validity of the International Classification of Functioning, Disability and Health (ICF) Comprehensive Core Set for osteoarthritis (OA) in order to test its possible use as a measuring tool for functioning.

**Methods:**

100 patients with OA (84 F, 16 M; mean age 63 yr) completed forms including demographic and clinical information besides the Short Form (36) Health Survey (SF-36^®^) and the Western Ontario and McMaster Universities Index of Osteoarthritis (WOMAC). The ICF Comprehensive Core Set for OA was filled by health professionals. The internal construct validities of "Body Functions-Body structures" (BF-BS), "Activity" (A), "Participation" (P) and "Environmental Factors" (EF) domains were tested by Rasch analysis and reliability by internal consistency and person separation index (PSI). External construct validity was evaluated by correlating the Rasch transformed scores with SF-36 and WOMAC.

**Results:**

In each scale, some items showing disordered thresholds were rescored, testlets were created to overcome the problem of local dependency and items that did not fit to the Rasch model were deleted. The internal construct validity of the four scales (BF-BS 16 items, A 8 items, P 7 items, EF 13 items) were good [mean item fit (SD) 0.138 (0.921), 0.216 (1.237), 0.759 (0.986) and -0.079 (2.200); person item fit (SD) -0.147 (0.652), -0.241 (0.894), -0.310 (1.187) and -0.491 (1.173) respectively], indicating a single underlying construct for each scale. The scales were free of differential item functioning (DIF) for age, gender, years of education and duration of disease. Reliabilities of the BF-BS, A, P, and EF scales were good with Cronbach's alphas of 0.79, 0.86, 0.88, and 0.83 and PSI's of 0.76, 0.86, 0.87, and 0.71, respectively. Rasch scores of BF-BS, A, and P showed moderate correlations with SF-36 and WOMAC scores where the EF had significant but weak correlations only with SF36-Social Functioning and SF36-Mental Health.

**Conclusion:**

Since the four different scales derived from BF-BS, A, P, and EF components of the ICF core set for OA were shown to be valid and reliable through a combination of Rasch analysis and classical psychometric methods, these might be used as clinical assessment tools.

## Background

Osteoarthritis (OA) is the most common chronic joint disease for middle- and old-aged individuals and is frequently associated with short- and long-term disabilities [1,2]. As such, a variety of scales are available for measuring functioning in osteoarthritis [3]. In order to better understand what each scale is measuring it is possible to catalogue the items in these scales according to the International Classification of Functioning, Disability and Health (ICF). The ICF, which is a bio-psychosocial framework for understanding the components of health and health-related states, describes functioning with a standard classification system in which functioning is an umbrella term encompassing all body functions, body structures, and activities and participation (i.e. positive functioning); similarly, disability serves as an umbrella term for impairments, activity limitations or participation restrictions (i.e. negative consequences) [4]. Body functions are the physiological functions of body systems whereas body structures are anatomical parts of the body. Impairments are problems in body function or structure such as a significant deviation or loss. Activity is the execution of a task or action by an individual and represents the individual perspective of functioning. Participation is involvement in a life situation and represents the societal perspective of functioning. The ICF lists environmental factors that interact with all these constructs. Personal factors are also indicated, but as yet are not defined in the ICF. Almost all of the existing Patient Reported Outcome scales used in OA assess impairments and activity limitations but rarely participation or environmental factors [5].

Thus the ICF classification comprises 1424 categories divided into the four components (body functions, body structures, activities and participation, environmental factors) [4]. Given the obvious difficulties of using such a comprehensive taxonomy in everyday clinical practice and research, ICF Core Sets, which are short lists of ICF categories relevant for specific conditions, have been developed. Currently, there are ICF core sets for various musculoskeletal conditions including OA [6]. Two previous studies have reported on the validation of the ICF Core Set for OA. In the first study, the content and the external construct validity of the Comprehensive Core Set was supported in a group of patients with knee OA [7]. The second study complemented the development of a Brief Core Set by comparing the categories of the Comprehensive Core Set that explained most of the variance of functioning and health [8].

The description of functioning based upon the ICF involves the rating of ICF categories with the ICF qualifiers which are numeric codes that specify the extent or the magnitude of functioning in that category, or the extent to which an environmental factor is a facilitator or barrier. Qualifier ratings across a number of ICF categories result in a potential ordinal profile. Consequently such an ordinal profile may provide a useful tool for evaluating healthcare interventions. The important question is whether it is possible to use this ordinal profile as a measurement instrument for an ICF component. Thus, this study aimed to investigate the reliability and construct validity of the ICF Comprehensive Core Set for Osteoarthritis as a potential measurement tool for functioning. To accomplish this aim, the scalability of components of this ICF core set was tested by both modern and classical psychometric methods.

## Methods

### Patients and setting

Data was collected in the Department of Physical Medicine and Rehabilitation at the Medical Faculty of Ankara University, Turkey. A total of 100 outpatients diagnosed as knee and/or hip OA according to the American College of Rheumatology criteria for the classification and reporting OA of knee and hip were included in the study [9,10]. Patients with concomitant uncontrolled or severe systemic diseases, any recent surgery that might affect their health status, and any cognitive impairment that would preclude participation in the study were excluded. The study was approved by the Ethical Committee of the Faculty of Medicine, Ankara University. All patients gave informed consent and the study was carried out in compliance with Helsinki Declaration.

### Assessment

The assessment included the administration of the ICF Comprehensive Core Set for OA, the Western Ontario and McMaster Universities Index of Osteoarthritis (WOMAC, V3.1) [11] and the Short Form-36 Health Survey v1.0 (SF-36^®^) [12]. The scoring of ICF Core Set for all patients was performed by the physical and rehabilitation medicine specialists who were trained in a structured one-day workshop organized by the researchers of the WHO ICF Collaborating Center at the Ludwig-Maximilian University in Munich. These specialists took part in the International Validation Studies of Core Sets and were experienced in the scoring system since they collected the data of many patients with various musculoskeletal conditions such as osteoarthritis, low back pain, rheumatoid arthritis and chronic widespread pain. The questionnaires WOMAC and SF-36 were either self-completed by patients or the assessors administered them to those who were illiterate. Sociodemographic (age, gender, educational level, employment status) and clinical data (disease duration, location, comorbidities) were also recorded.

The ICF Comprehensive Core Set for OA consists of 13 categories from the component Body functions (BF), 6 from the component Body Structures (BS), 19 from the component Activities and Participation (AP), and 17 from the component Environmental Factors (EF) [6]. A generic qualifier scale was used to evaluate the extent of a patient's problem in each of the ICF categories. The qualifier scale of the components BF, BS and AP have five response levels, ranging from 0 to 4: no/mild/moderate/severe/complete problem. The qualifier scale of the component EF has 9 response levels, ranging from -4 to +4. A specific environmental factor can be a barrier (-1 to -4), or a facilitator (1 to 4), or can have no influence (0) on the patient's life. If a factor has an influence, the extent of the influence (either positive or negative) can be coded as mild, moderate, severe, or complete. In addition, there are two other response options "8 (not specified)" and "9 (not applicable)" for all ICF categories.

The WOMAC is a disease-specific index developed for OA of the knee or hip [11]. It consists of 24 items in three subscales: pain (5 items), stiffness (2 items), and physical function (17 items). There are five response options for every question ('0' none, '1' mild, '2' moderate, '3' severe and '4' extreme) in Likert form. In this study, validated Turkish version of WOMAC [13] was used and the scores were presented as 0-10 for each WOMAC subscale after a normalization procedure [11,14]. The summation of equally weighted three subscales provided a single value for WOMAC total score, thus being 0-30.

Health-related quality of life (HRQoL) was evaluated using the SF-36 questionnaire [15]. It contains 36 items that measure perceived health in 8 scales, namely, physical functioning (PF), role-physical (RP), bodily pain (BP), general health (GH), vitality (V), social functioning (SF), role-emotional (RE), and mental health (MH), with higher scores (range 0-100) reflecting better perceived health. Additionally, two summary scores can be obtained; the Physical Component Summary (PCS) score and the Mental Component Summary (MCS) score. The Turkish version of the SF-36 was used in the study [16].

### Internal Construct Validity

The internal construct validity of the items of the ICF Core Set for OA, proposed as a scale for each ICF component, was tested by Rasch analysis. This is the formal testing of an assessment or a scale against a mathematical measurement model which defines how interval scale measurement can be derived from ordinal questionnaires [17-19]. This model assumes that the probability of a given respondent affirming an item is a logistic function of the relative distance between the item difficulty and the person ability on a linear scale. Thus, for example, in the case of mobility, the probability of a person affirming a (dichotomous) item about mobility is a logistic function of the relative distance between the level of mobility expressed by the item (the item difficulty), and the level of mobility of the person (the person ability). The model estimates person ability independent of the distribution of the population, and item difficulty independent of the person ability [20]. Master's partial credit model (PCM) which is an extension of the Rasch dichotomous model for polytomous (more than two response categories) items was used in this study [21].

The process of Rasch analysis is iterative, certain pathways are applied to each scale where an item set is intended to be summated to give a score. Initially, where polytomous items are involved, the response categories are examined for correct ordering. This is reflected by successive thresholds (point at which probability of being in adjacent thresholds is equal) demonstrating increasing levels of the construct being measured. The respondents' inconsistent use of response options can result in disordered thresholds and usually, in these circumstances, the collapsing of categories improves overall fit to the model [22].

Following this a range of tests are undertaken with respect to local dependency, probabilistic ordering (fit), unidimensionality and differential item functioning (DIF). The assumption of local independence implies that when the 'Rasch factor' has been extracted, that is, the main scale, there should be no leftover patterns in the residuals [23]. When a pair of items has a residual correlation of 0.20 or more than the average residual correlation, this is indicative of local response dependency between the items [24]. Such dependency inflates reliability as the items are, in practice, near replications of each other. This issue is dealt with by creating testlets - summary scores from the items that are locally dependent, which are then treated as one new larger variable [25]. Testlets were created considering the contents (*what they assess*) and response dependency of the items where mostly clinically relevant items were found to be locally dependent.

A variety of fit statistics are used to test if the data conform to Rasch model expectations. In the RUMM2030 programme [26], two are item-person interaction statistics transformed to approximate a z score, representing a standardized normal distribution. If the items and persons fit the model, these interaction statistics would have a mean of approximately zero and a standard deviation (SD) of one. A third summary statistic is a summed chi-square within groups defined by their position on the trait, where the overall chi-square for items is summed to give the item trait interaction statistic, testing the property of invariance across the trait. A significant chi-square indicates that the hierarchical ordering of the items varies across the trait, so compromising the required property of invariance. The significance of all chi-square fit statistics are Bonferroni adjusted to account for multiple testing [27]. In addition to these overall summary fit statistics, individual person- and item-fit statistics are presented, as (a) residuals (a summation of individual person and item deviations), (b) as a chi-square statistic, and (c) as an analysis of variance (ANOVA) with the residuals summed across the main effects of class intervals. Fit residuals between ± 2.5 are deemed to be adequate. These are summated within ability groups to provide the basis of the ANOVA analysis.

A formal test of the assumption of unidimensionality is undertaken by performing a principle component analysis (PCA) of the residuals. Items with the highest positive and negative correlations on the first residual factor are used to construct two smaller scales, anchored to the item difficulties of the main analysis [28]. The person estimates derived from these two subsets of items are then contrasted for each individual by a t test. A significant difference would be expected to occur by chance in 5% of the cases. Consequently, the percentage of tests outside the range ± 1.96 is reported, together with a 95% binomial confidence interval. This interval should overlap 5% for a non-significant finding to confirm unidimensionality.

Items are also tested for DIF. In the framework of Rasch measurement, the scale should be free of item bias or DIF [29]. DIF occurs when different groups within the sample (e.g., males and females), despite equal levels of the underlying characteristic being measured, respond in a different manner to an individual item. For example, men and women with equal levels of mobility may respond systematically differently to a mobility item such as walking 100 metres unaided. DIF can be detected both statistically and graphically. In the current analysis, DIF was tested by age, gender, years of education, and disease duration. The statistical test for DIF is an ANOVA, with main effects, for example for gender, and ability level. This examines the main effect for gender (uniform DIF) where any difference is constant across the trait. An interaction effect between ability level and the contextual factor under investigation (e.g. gender) identifies non-uniform DIF, where the difference between groups varies across the trait.

For item sets which constitute a potential new scale, all the above Rasch assumptions are considered together to determine which items are most suitable for retention. Poor items are removed, and the data refitted to the model until an adequate locally independent, unidimensional scale, free of DIF, is achieved. Finally the targeting and Person Separation Index (PSI) reliability of the scale are considered. A scale is perfectly targeted when the mean of the persons is the same as the mean of the items on their shared common metric. PSI is an estimate of internal consistency reliability and can be interpreted much the same as Cronbach's alpha, but has the linear transformation from the Rasch model substituted for the ordinal raw score [30].

### Reliability

Reliabilities of ICF components or proposed scales were initially tested by internal consistency which is an estimate of the degree to which its constituent items are interrelated, and is assessed by Cronbach's alpha coefficient [31]. Usually a reliability of 0.70 is required for analysis at the group level, and values of 0.85 and higher for individual use [32]. Subsequently reliability was further tested by the PSI from the Rasch analysis. Where the distribution is normal these two reliability indicators are equivalent, but where distributions are skewed, the PSI gives a more accurate indication of internal consistency reliability.

### External construct validity

External construct validity was determined by testing for expected associations of ICF components or proposed scales with WOMAC and SF-36 through the process of convergent construct validity [33]. In this study, the degree of associations was analyzed by Spearman's correlation coefficient.

### Sample size and statistical software

For the Rasch analysis, a sample size of 100 patients will estimate item difficulty, with α of 0.05, to within ± 0.5 logits [34]. Bonferroni correction was applied to both fit and DIF statistics due to the multiple testing [27]. Statistical analysis was undertaken with SPSS 11.5, Rasch analysis with RUMM2030 package [26].

## Results

### Patient characteristics

The mean age of the 100 patients was 62.9 ± 12.3 and 84% were female. The median education duration was 4.5 years (0-18 years). Ten percent of the patients was employed and the rest were either retired (20%) or housewives (70%). The median disease duration was 112 months (3-408 months). The WOMAC AND SF-36 scores of the patients are shown in Table 1.

**Table 1:** WOMAC and SF-36 scores of patients

Scales/subscales (n)	Mean ± SD	Median (Min-Max)
WOMAC-Pain (n = 95)	3.6 ± 2.2	3.5 (0-10)

WOMAC-Stiffness (n = 95)	2.9 ± 2.2	2.5 (0-7.5)

WOMAC-Physical function (n = 95)	4.0 ± 2.1	4.0 (0.1-9.8)

WOMAC-Total (n = 95)	10.5 ± 5.7	9.9 (0.1-26.1)

SF_Physical Functioning (n = 100)	48.8 ± 26.1	50 (0-100)

SF_Role-Physical (n = 100)	31.5 ± 41.7	0 (0-100)

SF_Bodily Pain (n = 100)	40.8 ± 20.4	35 (0-100)

SF_General health (n = 100)	44.6 ± 20.2	43.5 (5-100)

SF_Vitality (n = 100)	42.5 ± 24.6	40 (0-100)

SF_Social Functioning (n = 100)	56.9 ± 26.8	50 (0-100)

SF_Role-Emotional (n = 100)	41.0 ± 45.4	0 (0-100)

SF_Mental Health (n = 100)	57.4 ± 20.8	60 (12-100)

SF_Physical Health Component (n = 100)	35.7 ± 9.1	35.0 (16.8-61.0)

SF_Mental Health Component (n = 100)	41.3 ± 12.2	39.4 (18.4-70.4)

### Internal construct validity

#### "Body functions and body structures" (BF-BS) component

Initially we analysed the BF and BS items separately, but there was a problem with the 'sensation of pain' item (b280) which would have to be deleted. From a clinical point of view this was unacceptable, and so the potential of merging the two domains together was examined. Here we discovered that there was also local dependency across the 'b280 sensation of pain' and 's750 structure of the lower extremity' items. This suggested that in this group of people with hip and knee osteoarthritis, there may be some overlap between categories across domains. Consequently we merged the BF and BS items.

**Figure 1:**
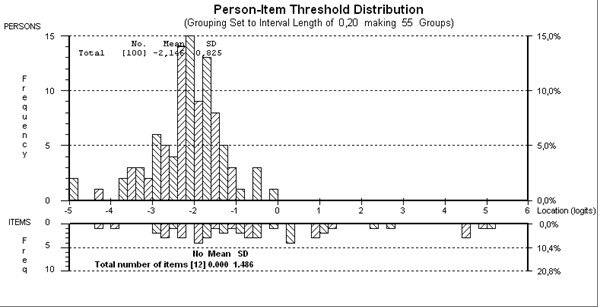
**Person-item threshold map of "Body functions and body structures" scale**.

Starting with 19 items, 10 "body functions" and 3 "body structures" categories displayed disordered thresholds, necessitating collapsing of response options. Following this, four testlets were created in order to overcome the problem of local dependency (testlet1: b280, s750; testlet2: s720, s730; testlet3: b715, b720, b760; testlet4: b730, b740, s740). After this modification, fourth testlet was removed due to the lack of fit. The remaining 16 items were found to fit the model (given a Bonferroni adjustment fit level of 0.003) (Table 2), with an overall mean item fit residual of 0.138 (SD 0.921) and mean person fit residual of -0.147 (SD 0.652). Item-trait interaction was non-significant, supporting the invariance of items (chi-square 40.83 (df = 24), p = 0.017). The PSI was good (0.76) indicating the ability of the scale to differentiate between 3 groups of patients. All items were free of DIF by age, gender, years of education and disease duration. Finally, using the PCA of residuals and obtaining two further estimates, no significant difference in person estimates (t = 9.0%; CI 4.7%-13.3%) was found between the two subsets, thus supporting the unidimensionality of the scale. Although mean person location of -2.146 was less than that of the items, indicating an offset of persons (i.e. less impairment) to the centre of the scale, the distribution of thresholds was wide enough to maintain the ability to statistically discriminate the respondents (Figure 1).

**Table 2:** Fit of the "Body functions and body structures" scale to Rasch model

ICF code	ICF category title	Location	SE	Individual Item Fit Residual	Chi-Square Test Statistics	p
Testlet1						
b280 s750	Sensation of pain Structure of lower extremity	-1.682	0.075	2.286	8.795	0.012

Testlet2						
s720 s730	Structure of shoulder region Structure of upper extremity	-0.723	0.098	0.735	3.481	0.175

Testlet3						
b715 b720 b760	Stability of joint functions Mobility of bone functions Control of voluntary movement functions	-0.408	0.109	0.463	1.208	0.547

b130	Energy and drive functions	-0.383	0.117	-0.611	5.219	0.074

b134	Sleep functions	-1.096	0.147	-0.673	3.947	0.139

b152	Emotional functions	-0.943	0.116	-0.207	1.773	0.412

b710	Mobility of joint functions	-1.274	0.125	-0.714	3.305	0.192

b735	Muscle tone functions	3.319	0.776	-0.710	1.882	0.390

b770	Gait pattern functions	0.030	0.205	0.224	7.326	0.026

b780	Sensations related to muscles and movement functions	0.370	0.162	0.083	0.153	0.926

s770	Additional musculoskeletal structures related to movement	0.425	0.190	1.207	0.112	0.946

s799	Structures related to movement, unspecified	2.365	0.346	-0.432	3.634	0.163

#### "Activities and participation" component

Although in the ICF, the domains in "Activities and Participation" are given as a single list and the components of "Activities" and "Participation" are not distinguished, it is also possible to designate some domains as activities and others as participation [4]. In this respect, the items related to activities and participation were analyzed separately. The items d410, d415, d430, d440, d445, d450, d455, d510, d530, d540, and d640 were designated to "Activities" and d470, d475, d620, d660, d770, d850, d910, and d920 to "Participation".

#### "Activities" (A) component

**Figure 2:**
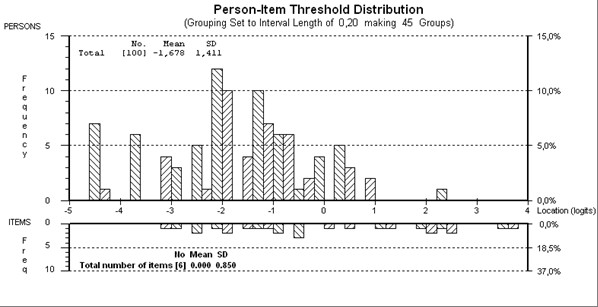
**Person-item threshold map of "Activity" scale**.

Starting with 11 items, five items displayed disordered thresholds, necessitating collapsing of categories. After creating two testlets (testlet1: d430, d440, d445; testlet2: d410, d415, d455) and removing testlet2 due to misfit to the model, an eight-item scale satisfied Rasch assumptions (given a Bonferroni adjustment fit level of 0.008) (Table 3), with an overall mean item fit residual of 0.216 (SD 1.237) and mean person fit residual of -0.241 (SD 0.894). Item-trait interaction was non-significant, supporting the invariance of items (chi-square15.67 (df = 12), p = 0.207). The PSI was good (0.86) indicating the ability of the scale to differentiate between 4 groups of patients. No significant difference was found in person estimates (t = 7.6%; CI 3.2%-12.1%) between two subsets, thus supporting the unidimensionality of the scale. All items were free of DIF. The mean person location of -1.678 was less than the average of the items indicating that the patients were more active than the average level of activity of the scale (Figure 2).

**Table 3:** Fit of the "Activity" scale to Rasch model

ICF code	ICF category title	Location	SE	Individual Item Fit Residual	Chi-Square Test Statistics	p
Testlet1						
d430 d440 d445	Lifting and carrying objects Fine hand use Hand and arm use	0.156	0.087	-0.235	0.471	0.790

d450	Walking	-0.799	0.143	2.069	5.327	0.070

d510	Washing oneself	-0.261	0.150	-0.831	4.122	0.127

d530	Toileting	1.216	0.167	1.482	0.907	0.635

d540	Dressing	0.667	0.221	-0.622	3.326	0.190

d640	Doing housework	-0.978	0.138	-0.566	1.519	0.468

#### "Participation" (P) component

**Figure 3:**
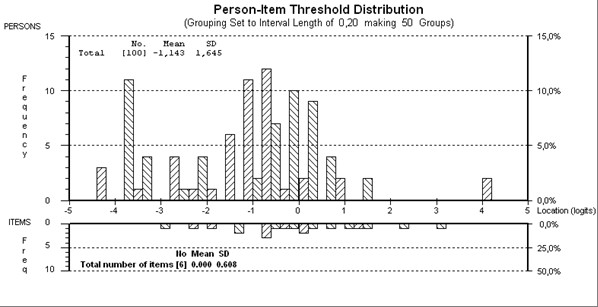
**Person-item threshold map of "Participation" scale**.

Starting with eight items, eight items displayed disordered thresholds, necessitating collapsing of categories. After creating one testlet from the items "d470 and d475" and removing d850 due to DIF by gender, the remaining seven items were found to fit the model (given a Bonferroni adjustment fit level of 0.008) (Table 4), with an overall mean item fit residual of 0.759 (SD 0.986) and mean person fit residual of -0.310 (SD 1.187). Item-trait interaction was non-significant, supporting the invariance of items (chi-square 15.88 (df = 12), p = 0.197). The PSI was good (0.87) indicating the ability of the scale to differentiate between 4 groups of patients. Finally, using the PCA of residuals and obtaining two further estimates, no significant difference in person estimates (t = 7.6%; CI 3.2%-12.1%) was found between the two subsets, thus supporting the unidimensionality of the scale. All items were free of DIF. The mean person location of -1.143 indicated that the patients were participating at a higher level than the average of the scale (Figure 3).

**Table 4:** Fit of the "Participation" scale to Rasch model

ICF code	ICF category title	Location	SE	Individual Item Fit Residual	Chi-Square Test Statistics	p
Testlet1						
d470 d475	Using transportation Driving	-0.288	0.283	2.001	7.227	0.027

d620	Acquisition of goods and services	-0.386	0.166	1.064	0.783	0.676

d660	Assisting others	-0.139	0.134	-0.807	4.221	0.121

d770	Intimate relationships	1.161	0.241	1.104	0.407	0.816

d910	Community life	-0.480	0.137	1.146	0.954	0.621

d920	Recreation and leisure	0.132	0.205	0.048	2.283	0.319

#### "Environmental Factors" (EF) component

While the original qualifier scale of the environmental factors ranged from -4 to +4, these 9 response levels do not represent a cumulative measurement of the impact of those environmental factors. As a result of this, barriers were rescored to 0, no influence was rescored to 1 and facilitators to 2.

**Figure 4:**
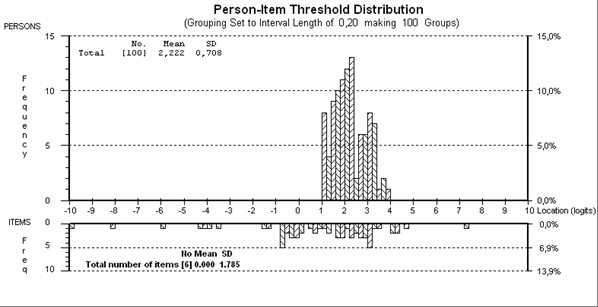
**Person-item threshold map of "Environmental factors" scale**.

Starting with the 17 items in the core set, 9 items displayed disordered thresholds, necessitating collapsing of response options. Following this, three testlets were created in order to overcome the problem of response dependency (testlet1: e120, e135, e150; testlet2: e310, e355, e410, e450, e540, e580; testlet3: e110, e225, e340, e460). After removal of the third testlet due to the lack of fit, fit to the model for the remaining thirteen-item scale was now satisfactory (given a Bonferroni adjustment fit level of 0.008) (Table 5). Overall mean item fit residual was -0.079 (SD 2.200) and mean person fit residual was -0.491 (SD 1.173). Item-trait interaction was non-significant, supporting the invariance of items (chi-square 15.73 (df = 12), p = 0.204). The PSI was good (0.71) indicating the ability of the scale to differentiate between 2 groups of patients. The unidimensionality of the scale was supported by the individual t-test showing 4.0% of tests as significant (CI -0.3%-8.3%). Given the mean person location was 2.222, this indicated that the majority of environmental factors experienced by the patients were facilitators (Figure 4). All items were free of DIF.

**Table 5:** Fit of the "Environmental factors" scale to Rasch model

ICF code	ICF category title	Location	SE	Individual Item Fit Residual	Chi-Square Test Statistics	p
Testlet1						
e120 e135 e150	Products and technology for personal indoor and outdoor mobility and transportation Products and technology for employment Design, construction and building products and technology of buildings for public use	1.316	0.136	0.888	1.156	0.561

Testlet2						
e310 e355 e410 e450 e540 e580	Immediate family Health professionals Individual attitudes of immediate family members Individual attitudes of health professionals Transportation services, systems and policies Health services, systems and policies	1.113	0.048	-3.935	3.269	0.195

e115	Products and technology for personal use in daily living	-0.112	0.106	-1.141	3.654	0.161

e155	Design, construction and building products and technology of buildings for private use	1.901	0.128	2.397	4.402	0.111

e320	Friends	-2.579	0.109	0.520	0.560	0.756

e575	General social support services, systems and policies	-1.639	0.130	0.796	2.688	0.261

### Reliability

Reliabilities of BF-BS, A, P, and EF were good with Cronbach's alpha values of 0.79, 0.86, 0.88, and 0.83 and, PSI's of 0.76, 0.86, 0.87 and 0.71, respectively.

### External construct validity

Associations of BF-BS, A, P, and EF scales with SF-36 and WOMAC are shown in Table 6. Correlations of BF-BS, A, and P scales with the SF-36 and the WOMAC were similar. The highest correlations (all of which at moderate level) were observed with SF36-Bodily pain, SF36-Social functioning, and WOMAC-Physical function subscales. The EF scale had significant but weak correlations only with SF36-Social Functioning and SF36-Mental Health.

**Table 6:** Correlations of BF-BS, A, P and EF scales with SF-36^® ^and WOMAC

	Rasch_BF-BS Scores	Rasch_A Scores	Rasch_P Scores	Rasch_EF Scores
SF-36_Physical Functioning	-0.447***	-0.361***	-0.414***	-0.011

SF-36_Role-Physical	-0.457***	-0.296**	0.392***	-0.044

SF-36_Bodily Pain	-0.580***	-0.573***	-0.606***	0.149

SF-36_General health	-0.452***	-0.422***	-0.478***	0.097

SF-36_Vitality	-0.371***	-0.286**	-0.338**	0.064

SF-36_Social Functioning	-0.498***	-0.547***	-0.578***	0.265**

SF-36_Role-Emotional	-0.320***	-0.352***	-0.455***	0.195

SF-36_Mental Health	-0.431***	-0.502***	-0.494***	0.242*

SF-36_Physical Health Component	-0.529***	-0.379***	-0.443***	-0.077

SF-36_Mental Health component	-0.392***	-0.410***	-0.480***	0.188

WOMAC_Pain	0.404***	0.247*	0.352***	0.005

WOMAC_Stiffness	0.354***	0.236*	0.267**	-0.002

WOMAC_Physical Function	0.583***	0.450***	0.587***	-0.048

WOMAC_Total	0.519***	0.353***	0.461***	-0.036

*: p < 0.05, **: p < 0.01, ***: p < 0.001

## Discussion

The ICF has become a widely accepted framework to describe functioning, disability and health from a bio-psycho-social perspective. Functioning and disability are at the centre of health care provision. In order to make the ICF applicable in healthcare, ICF Core Sets have been developed for specific diseases or conditions [35]. ICF Core Sets are selections of ICF categories relevant for a specific condition, which can be used in clinical studies or health statistics (brief ICF core sets) or to guide multidisciplinary assessments (comprehensive ICF core sets). For clinical practice and research, they list the ICF categories which should be measured but they provide no information about how to measure them. Although the ICF qualifier can be used to rate each ICF category, this provides an ordinal interpretation of the patient's level of functioning in various components of the ICF. Consequently, a scale with interval level measurement properties which would allow the calculation of change scores for each ICF component, would facilitate the use of ICF in health care setting.

The present study has investigated the scalability of components of the ICF Comprehensive Core Set for OA as potential measurement scales. It was done so by using a combination of classical methods such as convergent construct validity, and modern psychometric methods through Rasch analysis of the internal construct validity of the scales. After some modifications, the resulting BF-BS (16 items), A (8 items), P (7 items), and EF (13 items) scales derived from the Core Set were found to be reliable and valid. The modifications included firstly the collapsing of the categories of some of the items. Secondly, to overcome the local dependency problem some testlets were created according to the content and response dependency of the items and then tested for validity and reliability. The first modification is not uncommon for polytomous scales [36] but may indicate the need to reconsider the category structure if further studies encounter the same problem. The second (testlet) strategy is relatively recent in health outcome applications, but has shown to be influential in accommodating clinically important variations on the same theme while not compromising the integrity of the psychometric evaluation [37].

Nevertheless, the scales derived from the ICF Core Set for OA which fit to the Rasch model do not include all the items, some of which are indeed quite relevant to the component they are expected to assess. For example in BF-BS scale, items "b730 muscle power", "b740 muscle endurance functions", "s740 structure of pelvic region" had to be removed because of misfit. Thus, only "b735 muscle tone" and "b760 control of voluntary movement" remained in the resulting scale which might also be relevant to the muscle function.

In the activities component, the items "d410 changing basic body position", "d415 maintaining a body position", and "d455 moving around" which were united to form a testlet with respect to response dependency were removed because of misfit. In the participation items only "d850 remunerative employment" was removed since it displayed DIF for gender. This is quite relevant to the study population's demographics since the rate of employment is higher in men than that of women.

The need to rescore the environmental qualifiers remains an important issue. As they stand at the moment, the -4 to +4 scoring represents a single peak function, not a cumulative function as required for the Rasch model. In the latter, a score of +4 would indicate that all previous levels had also been affirmed (probabilistically so), while this is clearly not the case. To try and account for this, we created a simple three category response option which can be simply interpreted as the degree of facilitation, from less (0) to more (2). Empirically this appears to have been successful, although not before dichotomising many of the items, and so more attention will need to be given as to how such aspects should be scored. This same strategy was previously adopted in another study investigating the dimensionality of the ICF core set for low back pain using Rasch analysis [38].

There has been an earlier report which also investigated to construct a clinical measure of functioning by integrating the ICF categories in OA [39]. However the methodology used in that study differed from our study such that they did not specifically examine modern tests of unidimensionality, nor did they examine local dependency, but rather created an item bank including BF, BS and AP items. In the present study, the Rasch strategy used was focused on developing robust scales out of the items of the components of the ICF core set for OA which satisfied all the assumptions of the Rasch model.

The scales derived from the ICF OA core set in the current study were found to be reliable in terms of internal consistency and PSI by the Rasch analysis. However, as the rating of ICF categories is an assessor dependant evaluation inter-rater reliability testing should also have been performed. This is an important issue as inter-rater reliability has been reported to be relatively low in another study investigating reliability of ICF core set for RA [40]. Also, the level of reliability for the Body Structures and Functions set displayed reliability only consistent with group use. Thus the current sample had relatively low levels of impairment, and this skewed distribution may have affected the level of reliability.

The external construct validity of the scales derived from the ICF Core Set components were tested by associations with two outcome measures, the WOMAC and the SF-36. The BF-BS, A and P scales showed only moderate correlations with both measures. This was expected as only a few categories in BF-BS set and half of the categories in A and P sets were linked to the items of those measures [5,41]. Also, as expected, the EF set showed no associations with the total scores of WOMAC and the SF-36 since none of the EF categories were found to be linked to both measures [5,41].

The study raises a number of issues with regard to the structure of the ICF classification, particularly the separation of activities and participation. In the current study we adopted the first method of classification highlighted in the ICF (4). This is where some domains are specified as activities, and others as participation, without overlap. Unfortunately there is very little agreement in the literature as to which domains belong to which component, and thus a variety of solutions have been put forward in recent times [42-44]. The tests of strict unidimensionality which have been used to support the items within our choice of domains for each component suggest that this choice offers at least one potential solution to the separation of the components.

There are a number of limitations to the study. The sample size is small, and only gives a degree of precision to item and person location within 0.5 logits. Given the Rasch model allows an adaptation to interval scaling, then a nonogram giving the exchange rate between the raw score and latent interval scale estimate would have been useful. However, this does require a larger sample size (e.g. 250 cases or 20 times the number of items, whichever is the larger) and so will have to wait until larger replications are undertaken. The collapsing of categories also impedes the production of the exchange rate as this will require further evidence and consensus of scoring options. Thus the evidence relating to the qualifiers can at best be considered provisional until repeated evidence on larger samples support the current interpretation.

In the present study, in order to get a rather homogenous population in a limited number of patients, only patients with knee and/or hip OA were analyzed. Therefore the scales proposed and tested here can only be used for this specific OA group, not for other types of involvement such as hand or spine OA. Therefore, these results should be replicated in larger samples including all types of OA. However, the results of this study do demonstrated the potential of the ICF core set for OA as a scale, despite the limitation mentioned above.

## Conclusions

The four different scales derived from BF-BS, A, P, and EF components of the ICF core set for OA were shown to be valid and reliable through Rasch analysis and classical psychometric methods. These scales should further be tested in larger samples, including cross-cultural validity evaluation, given the ICF is intended to be used as an international classification.

## Competing interests

The authors declare that they have no competing interests.

## Authors' contributions

YK, DG, AAK, SK, and AT all contributed to the trial concept and design, interpretation of data and drafting of the manuscript. DG and AT undertook the statistical analysis. YK, AAK, and MH conducted all data collection. All authors revised the manuscript for important intellectual content and have read and approved the final version.

## Pre-publication history

The pre-publication history for this paper can be accessed here:

http://www.biomedcentral.com/1471-2474/12/255/prepub

## References

[B1] Reginster JY The prevalence and burden of arthritis Rheumatology (Oxford) 2002 41 Suppl 1 3 6 12173279

[B2] Botha-Scheepers S Riyazi N Kroon HM Scharloo M Houwing-Duistermaat JJ Slagboom E Rosendaal FR Breedveld FC Kloppenburg M Activity limitations in the lower extremities in patients with osteoarthritis: the modifying effects of illness perceptions and mental health Osteoarthritis Cartilage 2006 14 1104 1110 10.1016/j.joca.2006.04.011 16740397

[B3] Pollard B Johnston M The assessment of disability associated with osteoarthritis Curr Opin Rheumatol 2006 18 531 536 10.1097/01.bor.0000240368.39713.e6 16896296

[B4] World Health Organization International Classification of Functioning, Disability, and Health: ICF 2001 Geneva

[B5] Weigl M Cieza A Harder M Geyh S Amann E Kostanjsek N Stucki G Linking osteoarthritis-specific health-status measures to the International Classification of Functioning, Disability, and Health (ICF) Osteoarthritis Cartilage 2003 11 519 523 10.1016/S1063-4584(03)00086-4 12814615

[B6] Dreinhöfer K Stucki G Ewert T Huber E Ebenbichler G Gutenbrunner C Kostanjsek N Cieza A ICF core sets for osteoarthritis J Rehabil Med 2004 44 Suppl 75 80 15370752 10.1080/16501960410015498

[B7] Xie F Lo NN Lee HP Cieza A Li SC Validation of the Comprehensive ICF Core Set for Osteoarthritis (OA) in patients with knee OA: a Singaporean perspective J Rheumatol 2007 34 2301 2307 17937460

[B8] Xie F Lo NN Lee HP Cieza A Li SC Validation of the International Classification of Functioning, Disability, and Health (ICF) Brief Core Set for osteoarthritis Scand J Rheumatol 2008 37 450 461 10.1080/03009740802116216 18666026

[B9] Altman R Alarcon G Appelrouth D Bloch D Borenstein D Brandt K The American College of Rheumatology criteria for the classification and reporting of osteoarthritis of the hip Arthritis Rheum 1991 34 505 514 10.1002/art.1780340502 2025304

[B10] Altman R Asch E Bloch D Bole G Borenstein D Brandt K Christy W Cooke TD Greenwald R Hochberg M Development of criteria for the classification and reporting of osteoarthritis. Classification of osteoarthritis of the knee. Diagnostic and Therapeutic Criteria Committee of the American Rheumatism Association Arthritis Rheum 1986 29 1039 1049 10.1002/art.1780290816 3741515

[B11] Bellamy N Buchanan WW Goldsmith CH Campbell J Stitt LW Validation study of WOMAC: a health status instrument for measuring clinically important patient relevant outcomes to antirheumatic drug therapy in patients with osteoarthritis of the hip or knee J Rheumatol 1988 15 1833 1840 3068365

[B12] Ware JJ Sherbourne CD The MOS 36-item short-form health survey (SF-36). I. Conceptual framework and item selection Medical Care 1992 30 473 483 10.1097/00005650-199206000-00002 1593914

[B13] Tüzün EH Eker L Aytar A Daşkapan A Bayramoğlu M Acceptability, reliability, validity and responsiveness of the Turkish version of WOMAC osteoarthritis index Osteoarthritis Cartilage 2005 13 28 33 10.1016/j.joca.2004.10.010 15639634

[B14] Kersten P White PJ Tennant A The visual analogue WOMAC 3.0 scale-internal validity and responsiveness of the VAS version BMC Musculoskelet Disord 2010 30 80 10.1186/1471-2474-11-80 PMC2874767 20433732

[B15] SF-36 http://www.sf-36.org/

[B16] Kocyigit H Aydemir O Fisek G Olmez N Memis A Kisa form-36 (KF-36)'nin Türkçe versiyonunun güvenilirligi ve geçerliliği. Romatizmal hastaliği olan bir grup hasta ile çalisma Ilaç ve Tedavi Dergisi 1999 2 102 10622040296

[B17] Rasch G Probabilistic models for some intelligence and attainment tests 1960 Chicago: University of Chicago Press

[B18] Luce RD Tukey JW Simultaneous conjoint measurement: A new type of fundamental measurement J Math Psychol 1964 1 1 27 10.1016/0022-2496(64)90015-X

[B19] Newby VA Conner GR Grant CP Bunderson CV The Rasch model and additive conjoint measurement J Appl Meas 2009 10 348 354 19934524

[B20] Andrich D Rasch models for measurement 1988 London: Sage Publications

[B21] Masters G A Rasch model for partial credit scoring Psychometrica 1982 47 149 174 10.1007/BF02296272

[B22] Tennant A Conaghan PG The Rasch Measurement Model in Rheumatology: What is it and why use it? When should it be applied, and what should one look for in a Rasch paper? Arthritis Rheum 2007 57 1358 1362 10.1002/art.23108 18050173

[B23] Reeve BB Hays RD Bjorner JB Cook KF Crane PK Teresi JA Thissen D Revicki DA Weiss DJ Hambleton RK Liu H Gershon R Reise SP Lai JS Cella D PROMIS Cooperative Group Psychometric evaluation and calibration of health-related quality of life item banks: plans for the Patient-Reported Outcomes Measurement Information System (PROMIS) Med Care 2007 45 Suppl 1 22 31 10.1097/01.mlr.0000250483.85507.04 17443115

[B24] Marais I Andrich D Formalising dimension and response violations of local independence in the unidimensional Rasch model J Applied Measurement 2008 9 200 215 18753691

[B25] Wainer H Kiely GL Item clusters and computer adaptive testing: A case for testlets J Educ Meas 1987 24 185 210 10.1111/j.1745-3984.1987.tb00274.x

[B26] Andrich D Lyne A Sheridan B Luo G RUMM 2030 2009 Perth: RUMM Laboratory

[B27] Bland JM Altman DG Multiple significance tests: the Bonferroni method BMJ 1995 310 170 10.1136/bmj.310.6973.170 7833759 PMC2548561

[B28] Smith EV Jr Detecting and evaluating the impact of multidimensionality using item fit statistics and principal component analysis of residuals J Appl Meas 2002 3 205 231 12011501

[B29] Teresi JA Kleinman M Ocepek-Welikson K Modern psychometric methods for detection of differential item functioning: application to cognitive assessment measures Stat Med 2000 19 1651 1683 10.1002/(SICI)1097-0258(20000615/30)19:11/12<1651::AID-SIM453>3.0.CO;2-H 10844726

[B30] Fisher WP Reliability statistics Rasch Measure Trans 1992 6 238

[B31] Cronbach LJ Coefficient alpha and the internal structure of tests Psychometrika 1951 16 297 334 10.1007/BF02310555

[B32] Nunally JC Bernstein IH Psychometric Theory 1994 Third New York: McGraw-Hill

[B33] Nunally JC Psychometric Theory 1978 New York: McGraw-Hill

[B34] Linacre JM Sample size and item calibration stability Rasch Measure Trans 1994 7 28

[B35] Cieza A Ewert T Ustün TB Chatterji S Kostanjsek N Stucki G Development of ICF Core Sets for patients with chronic conditions J Rehabil Med 2004 44 Suppl 9 11 15370742 10.1080/16501960410015353

[B36] Elhan AH Küçükdeveci AA Tennant A Pavia FF The Rasch Measurement Model Advances in Rehabilitation. Research Issues in Physical & Rehabilitation Medicine 2010 Italy: Maugeri Foundation 89 10221638887

[B37] Ndosi M Tennant A Bergsten U Kukkurainen ML Machado P Torre-Aboki JD Viet Vieland TP Zangi HA Hill J Cross-cultural validation of the Educational Needs Assessment Tool in RA in 7 European countries BMC Musculoskelet Disord 2011 12 110 10.1186/1471-2474-12-110 21609481 PMC3126763

[B38] Røe C Sveen U Geyh S Cieza A Bautz-Holter E Construct dimensionality and properties of the categories in the ICF Core Set for low back pain J Rehabil Med 2009 41 429 37 10.2340/16501977-0368 19479155

[B39] Cieza A Hilfiker R Chatterji S Kostanjsek N Ustün BT Stucki G The International Classification of Functioning, Disability, and Health could be used to measure functioning J Clin Epidemiol 2009 62 899 911 10.1016/j.jclinepi.2009.01.019 19540718

[B40] Uhlig T Lillemo S Moe RH Stamm T Cieza A Boonen A Mowinckel P Kvien TK Stucki G Reliability of the ICF Core Set for rheumatoid arthritis Ann Rheum Dis 2007 66 1078 1084 10.1136/ard.2006.058693 17223659 PMC1954720

[B41] Cieza A Stucki G Content comparison of health-related quality of life (HRQOL) instruments based on the international classification of functioning disability and health Qual Life Res 2005 14 1225 1237 10.1007/s11136-004-4773-0 16047499

[B42] Badley EM Enhancing the conceptual clarity of the activity and participation components of the International Classification of Functioning, Disability, and Health Social Sci Med 2008 66 2335 2345 10.1016/j.socscimed.2008.01.026 18314239

[B43] Whiteneck G Dijkers MP Difficult to Measure Constructs: Conceptual and Methodological Issues Concerning Participation and Environmental Factors Arch Phys Med Rehabil 2009 90 11 Suppl 1 S22 35 19892071 10.1016/j.apmr.2009.06.009

[B44] Dijkers MP Issues in the Conceptualization and Measurement of Participation: An Overview Arch Phys Med Rehabil 2010 91 9 Suppl 1 S5 16 20801280 10.1016/j.apmr.2009.10.036

